# Mandatory implementation of NICE Guidelines for the care of bipolar disorder and other conditions in England and Wales

**DOI:** 10.1186/s12916-015-0464-7

**Published:** 2015-09-30

**Authors:** Richard Morriss

**Affiliations:** Psychiatry and Community Mental Health, University of Nottingham, Nottingham, UK

**Keywords:** Bipolar disorder, Clinical guideline, Compulsion, Implementation, National Institute of Healthcare and Clinical Excellence (NICE), Organisation of care, Policy, Professional responsibility

## Abstract

**Background:**

Bipolar disorder is a common long-term mental health condition characterised by episodes of mania or hypomania and depression resulting in disability, early death, and high health and society costs. Public money funds the National Institute of Healthcare and Clinical Excellence (NICE) to produce clinical guidelines by systematically identifying the most up to date research evidence and costing its main recommendations for healthcare organisations and professionals to follow in England and Wales. Most governments, including those of England and Wales, need to improve healthcare but at reduced cost. There is evidence, particularly in bipolar disorder, that systematically following clinical guidelines achieves these outcomes.

**Discussion:**

NICE clinical guidelines, including those regarding bipolar disorder, remain variably implemented. They give clinicians and patients a non-prescriptive basis for deciding their care. Despite the passing of the Health and Social Care Act in 2012 in England requiring all healthcare organisations to consider NICE clinical guidelines in commissioning, delivering, and inspecting healthcare services, healthcare organisations in the National Health Service may ignore them with little accountability and few consequences. There is no mechanism to ensure that healthcare professionals know or consider them. Barriers to their implementation include the lack of political and professional leadership, the complexity of the organisation of care and policy, mistrust of some processes and recommendations of clinical guidelines, and a lack of a clear implementation model, strategy, responsibility, or accountability. Mitigation to these barriers is presented herein.

**Summary:**

The variability, safety, and quality of healthcare might be improved and its cost reduced if the implementation of NICE clinical guidelines, such as those for bipolar disorder, were made the minimum starting point for clinical decision-making and mandatory responsibilities of all healthcare organisations and professionals.

## Background

Bipolar disorder is a serious mental illness and a long-term condition characterised by recurrent episodes of mania or hypomania (elated mood, overactivity, disinhibition, and inflated self-esteem lasting at least 4 days) and depression lasting for 2 weeks interspersed by periods of being well or less severe symptoms [1]. Worldwide, among all health conditions, it is the 18th leading cause of years lost due to disability [2], with a peak age of onset between 13 and 30 years of age [3]. Standardised mortality ratios for all causes, cardiovascular disease and respiratory disease and suicide are all increased when coupled to the condition [4]. Acute mania is treated with antipsychotic drugs that differ in their clinical effectiveness and acceptability [5]. Acute bipolar depression is usually treated with a range of medication, while lithium remains one of the main approaches to long-term management [6]. There is accumulating evidence for the effectiveness of psychological treatments for bipolar depression and long-term management [7]. Some of the changes to practice proposed in the National Institute of Healthcare and Clinical Excellence (NICE) Clinical Guideline for Bipolar Disorder in 2014 [8] challenge long-standing clinical practice, e.g. antidepressants other than fluoxetine with olanzapine are not recommended to treat acute bipolar depression. The new clinical guideline explicitly promotes collaborative clinical practice with patients and carers based on recovery goals.

NICE was formed in 1999 by the United Kingdom government to develop national standards of healthcare to reduce variation in clinical care across different parts of England and Wales, but not Scotland and Northern Ireland, where the devolved government sets health policy. NICE now produces different types of guidance: clinical guidelines for healthcare, public health guidelines, social care guidelines, technology appraisal, and guidance on interventional procedures, medical technologies, and diagnostic agents [9]. Only some technology appraisals are mandatory for implementation by publicly funded healthcare organisations, namely the National Health Service (NHS) in England and Wales. Clinical guidelines consider the whole care pathway for a condition from primary care to secondary care. NICE Quality Standards [10] (Box 1) are specific measurable statements derived principally from the NICE clinical guidelines when practice is variable, but the evidence base for recommendations from the guideline is robust. They are auditable for quality improvement so that commissioners and services can demonstrate whether practice has improved. Neither clinical guidelines nor quality standards are mandatory for implementation by healthcare organisations or health professionals. It is the author’s view that they should be the starting point for all clinical care and, with respect to all recommendations concerning safety, the minimum standard of care expected in the NHS. Their implementation should be a mandatory responsibility for all healthcare commissioners, providers, inspection bodies, health professionals, and professional bodies responsible for licensing, registration, training, and revalidation. Otherwise, access to high standards of practice will remain variable, patients and carers are not empowered because they do not know what to expect, and care is less clinically safe, effective, and cost-effective than it should be.

### The case for the mandatory implementation of NICE guidelines

**Fig. 1:**
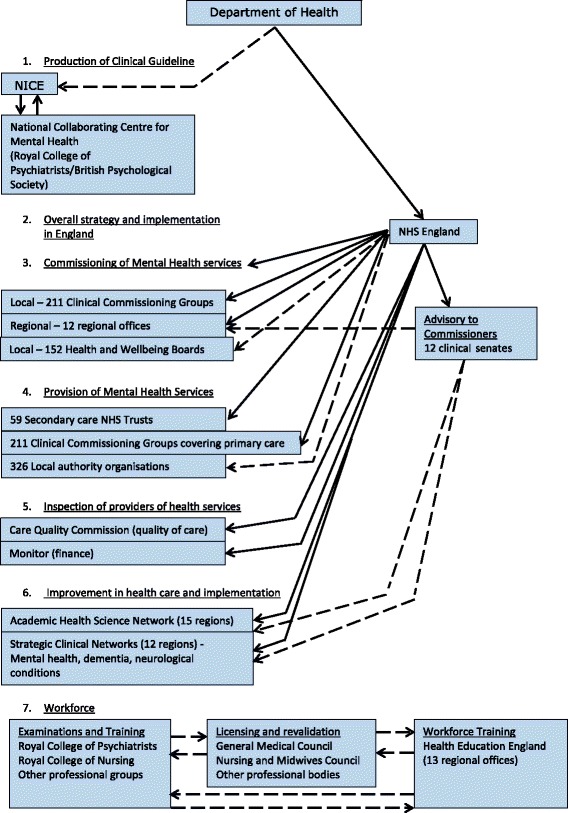
Relationships of NICE to National Health Services and Workforce organisations under Health and Social Act (2012). Indirect relationships Direct relationships. Does not fully represent all social care, public health, third sector, and independent contractors. England only. Wales is not represented

Box 2 summarises the potential benefits of the mandatory implementation of NICE clinical guidelines. Legislation was passed to promote the consideration of NICE clinical guidelines for health conditions by all NHS organisations in England under the Health and Social Care Act (2012) [11], and set up a number of other bodies such as Academic Health Science Networks and strategic clinical networks to help implement evidence-based care and innovation, including NICE clinical guidelines, into practice (Fig. 1). NICE clinical guidelines are best considered as a starting point for clinical care and are not designed to cover every clinical situation that may arise, so health professionals and NHS organisations must use their judgement to optimise clinical care for each patient they see [12]; these may be useful in other countries who wish to adopt the approach [13, 14]. Like most NICE clinical guidelines, the 2006 NICE Clinical Guideline for Bipolar Disorder [15] seems to have been incompletely and variably implemented [16]. For instance, the 2006 NICE Clinical Guideline for Bipolar Disorder recommendations concerning lithium monitoring were followed in 48–70 % patients in a recent national audit [17], while counselling about teratogenic risk and contraception in women of childbearing age taking anticonvulsants was only 22 % [18]. There is great variation in the implementation of NICE guidelines not only between organisations but also within the same organisation over time [19, 20]. In particular, NICE recommendations that require changes in the organisation of care or are conflicting to established practice are poorly implemented [16]. A more standardised and systematic approach to care as outlined by the NICE clinical guideline that is still patient centred should reduce one source of variability of outcome in patients with bipolar disorder, a condition with a highly variable outcome due to its natural history.

The same variable implementation of the NICE Clinical Guideline for Bipolar Disorder may be repeated with the 2014 update, despite the Health and Social Care Act (2012), because although NICE clinical guidelines must “*be carefully considered when developing strategies, planning services and prioritising resources*”, “*commissioners reserve the right not to implement NICE clinical guidelines*” [21], as do any other healthcare organisations or healthcare professionals, whether they are junior staff taking professional examinations or senior health professionals who are consultants in the topic of the clinical guideline facing appraisal and revalidation. It is unclear who makes these decisions in each healthcare body and there seem to be no lines of accountability or transparency in their decision-making. NICE clinical guidelines are often not considered in local commissioning of healthcare unless public health clinicians and more senior commissioners or national organisations are involved [22, 23]. A striking example of the consequences relates to the implementation of national guidance on self-harm and suicide, which includes NICE guidelines on self-harm, depression, and dual diagnosis (serious mental illness including bipolar disorder and alcohol or drug misuse) [24–26], as well as other policies [27]. Suicide rates fell between 1997 and 2006 in those localities where key national recommendations (24-hour crisis resolution and home treatment teams, dual diagnosis teams, and multidisciplinary review of suicide and other serious incidents) have taken place compared to no change or rises in suicide rates in those localities where these key recommendations were not implemented [28].

Failure of a NHS organisation to implement NICE clinical guidelines ought to be a rare decision that should be publicly justified given that NICE clinical guidelines are carefully costed and evidence based at a time when the NHS has been handed the task of improving quality of care whilst at the same time saving £30 billion by 2020 [29]. NICE clinical guidelines are developed after a thorough systematic review and meta-analysis of the evidence from research, economic modelling of key recommendations in the NICE guideline, and extensive iterative consultation with service users, carers, a multidisciplinary group of primary and secondary care clinicians, iteration with national and international academic experts, and iteration with national organisations [30]. Similar input goes into the development of care pathways, information for the public, and quality standards to provide a suite of tools that mental health services might utilise to help implement NICE guidance through audit and quality improvement.

Knowledge and practice in line with NICE clinical guidelines is usually required for good or outstanding ratings of the effectiveness of care when providers of health services are inspected by the Care Quality Commission [31]. Surprisingly, satisfactory care delivered by healthcare organisations in England does not necessarily require the demonstration of knowledge and practice in line with NICE clinical guidelines even though they cover patient safety, equity of access to care, and clinical and cost effectiveness. There have been major failings in the delivery of care to NHS organisations as outlined in the Francis report [32] and other subsequent reports; while the mandatory implementation of NICE clinical guidelines was not a specific recommendation of the Francis report, more robust support of its guidance and specifically its quality standards was endorsed by NICE, NHS England, and the Care Quality Commission (recommendation 11), but undermined by national commissioning policy in the same year [21].

When implemented systematically in a care pathway, NICE clinical guidelines may reduce the cost of care without worsening clinical outcomes [33]; however, there is a surprising lack of conclusive evidence on this issue. Importantly, a recent randomised controlled trial showed that guideline care involving a specialist bipolar disorder pathway using British Association of Psychopharmacology and NICE recommended treatments showed improved clinical outcomes at two thirds the cost compared to usual care by a community mental health team over 6 years [34]. Better care for no extra costs has been achieved before for bipolar disorder and schizophrenia when clinical guidelines have been followed [35]. There is also some evidence that better evidence-based quality of care is not only cost effective but cheaper [36, 37] because mistakes and poor outcomes require additional care.

### The case against mandatory implementation of NICE clinical guidelines

Box 3 summarises the potential harms resulting from the mandatory implementation of NICE clinical guidelines. A key issue is that the guideline accurately reflects the balance of evidence and in particular is cautious about recommendations that are very directive (i.e. “offer” or “do not do” should be used only in the face of compelling evidence versus “consider”), otherwise care may be misguided, wasteful, or harmful [38]. Clinical guidelines for bipolar disorder and other conditions vary considerably in what they recommend, although such variation may not matter provided the guideline is used only as a starting point for clinical decision-making rather than constraint of patient-centred care [39, 40]. Despite efforts to achieve objectivity, consensus among clinicians, patients, and carers, and transparency of decision-making [12], criticism of NICE guideline recommendations remain. For instance, the recent NICE guideline on psychosis and schizophrenia [41] has been criticised because its recommendations on cognitive behaviour therapy for schizophrenia became more directive despite no updated review of research evidence since 2009 [42]. An issue of central importance is therefore more frequent review by NICE of its evidence base for its recommendations, perhaps by an ongoing meta-analysis and a standing committee to review NICE clinical guidelines if there is sufficient evidence to potentially overturn recommendations and issue an update to the guideline. Furthermore, NICE clinical guidelines may be seen as over-reliant on the results of randomised controlled trials that often exclude most of the patients that the guideline would be applied to and extrapolate average group effects to individuals. Some critics would argue that randomised controlled trials should not be accepted as evidence unless they are complemented by evidence from routine clinical practice such as multi-centre observational cohort studies [43].

While there is evidence particularly in mental health that NICE guideline care improves recovery and service user experience [44–46], such care may not be sufficient to improve clinical outcomes [47]. Over-adherence to NICE guidelines can be associated with a lack of patient-centred care, poor clinical outcomes, and poor service user experience unless clinicians use their judgement and listen to patient preferences when they apply NICE guideline care [48, 49]. For these reasons, NICE clinical guidelines tend to outline factors for the clinician to consider and are not and should not be prescriptive, allowing care to be tailored to individual patient needs and preferences [12].

While NICE clinical guidelines might reduce variability in care, they may raise expectations of health professionals beyond their ability to deliver care given financial and other constraints, inform articulate rather than disadvantaged patients on how to obtain the care they want thus increasing health inequalities, and restrict health professionals from personalising care when necessary [50], particularly in the face of other mental and physical comorbidities [51]. NICE clinical guidelines contain a mixture of aspirational or developmental and mature recommendations; these may need clearer justification and separation in the NICE clinical guidelines between aspirational or development, and mature recommendations, plus investment and a timetable for implementation from NHS England for key recommendations that require investment. Mandatory implementation of NICE guidelines might be seen to discourage the development and uptake of new technologies in the NHS. However, the issue is complex [52]. Some technology would enable NICE clinical guideline care to be delivered more widely, e.g. mobile phone apps enabling self-completion of standardised measures of severity of depression [53]. The biggest problem for technology companies in working with the NHS is the complexity and unpredictability of decision-making on commissioning and utilisation of technology within it [54]. National clinical guidelines and processes may provide clarity and greater certainty, improving market conditions for the development, uptake, and sustainability of technology and other innovation within the NHS [54].

### Barriers and drivers to implementation of NICE guidelines for bipolar disorder

Table 1 summarises barriers and drivers to the implementation of NICE clinical guidelines for bipolar disorder. The Health and Social Care Act (2012) required all NHS organisations to consider NICE clinical guidelines in all commissioning and service delivery, but there has been a collective lack of leadership by political, professional, managerial, or patient advocates so they remain largely unimplemented. There may still be doubt over the wisdom of enforcing NICE clinical guideline care because of the fear of unintended consequences. Some NICE clinical guidelines encounter professional resistance, particularly over some recommendations. For instance, NICE Clinical Guideline 58 on prostate cancer made five recommendations that were met with widespread professional disagreement according to a national survey in 2008 although 60 % agreed the guideline as a whole would improve patient care. Two years later, two of these five recommendations received widespread support, two had the same level of professional disagreement, and one had even less support [55]. Some members of professions do not value evidence-based medicine, relying on other sources of evidence, such as peer group experience, to drive their decision making [56]. However, on the whole, NICE clinical guideline care is more likely to obtain professional support than other policy at a national and local level [22].

**Table 1:** Barriers to mandatory implementation of NICE clinical guidelines for bipolar disorder and their mitigation

Type of barrier	Nature of barrier	Mitigation against barrier
Policy	a) Lack of political, managerial, and professional leadership in mandating their implementation versus contest and ignorance of clinical guidelines	Affirmation of importance of implementation of NICE clinical guidelines unless there is a compelling reason not to by leaders
	b) Complexity of policy directed towards health and social care including mental health	Consider rationalisation of policy; obligation by NHS England, NICE, and professional bodies to ensure compatibility of existing policy with NICE clinical guidelines
Organisation of care	a) Multitude of NHS professional and social care bodies with overlapping roles, responsibilities, and differing or unclear lines of accountability	Consider rationalisation of organisation of care; require all agencies to focus on implementation of NICE guidelines with other agencies to improve effectiveness and efficiency of clinical pathways in line with NICE clinical guidelines
	b) Concern over professional and personal conflict of interest in development of NICE clinical guidelines, lack of psychiatric involvement because of pharmaceutical industry conflict of interest, and insufficient professional and NHS organisational engagement	Improve processes of developing clinical guidelines in line with Institute of Medicine’s recommendations to obtain full multidisciplinary professional, service user, and NHS input into NICE clinical guidelines, and manage any conflict of interest
Education	NICE clinical guidelines are low priority for training, licensing, continuing professional development, appraisal, and revalidation by professional and NHS workforce organisations	Affirm that principles, e.g. recovery and content of NICE clinical guideline care, are of central importance and design systems to ensure they are mandatory for training, examination, licensing, appraisal, continuing professional development, and revalidation
Economic	a) Some high cost items recommended in NICE clinical guidelines or innovation, e.g. technology; service redesign to improve care requiring investment with later cost offset	NHS England with other bodies, e.g. Academic Health Science Networks (AHSN), work with NICE to set timetable for implementation with non-recurrent funding for set up costs
	b) Guideline may discourage innovation and research by setting out specific recommendations for care	Guideline highlights areas of uncertainty for innovation and research
	c) Overall uncertainty about costs, benefits, unintended consequences, and harms with mandatory implementation of NICE clinical guidelines	Overall research and monitoring study commissioned with review dates to consider results and mitigating action
Treatment	Professionals will over rigidly apply or not conform to NICE clinical guidelines	Monitoring of NICE quality standards and service user experience as routine requirement of commissioning, inspection of providers, professional appraisal, and revalidation
Service user	Lack of knowledge of public about NICE guidance	Requirement of all NHS providers and AHSN to work with NICE to disseminate patient versions of NICE clinical guidelines and how to use them

The legitimacy of a clinical guideline and mistrust of it can arise when an organisation developing the guideline or key members of the committee or panel devising the guideline have conflicts of interest raising concerns about bias in favour of the interests [57]. In the United States, a survey found that 71 % of chairs of clinical policy committees had financial conflicts of interest while 84 % of doctors have expressed concern about industry influence over clinical guidelines [58]. NICE itself is publicly funded as a non-departmental public body of the Department of Health in the United Kingdom. NICE requires all conflicts of interest of members of clinical guideline committees to be reported before appointment and at each meeting; any member removes themselves from any decision that might be compromised by a conflict of interest. However, there are also concerns about other potential conflicts of interest such as the promotion of professional disciplinary issues and individual or groups of individuals’ professional interests on NICE clinical guideline development groups. Recently, NICE has started to appoint chairs of committees who are not experts in the condition under review; however, there is a danger that the richness of discussions at clinical guideline meetings is diluted with the production of an inferior guideline if all professionals with a conflict of interest are excluded from clinical guideline committees [59].

Responsibility for the implementation of NICE clinical guidelines is shared according to the Health and Social Care Act (2012) across a complex network involving over 620 different professional, healthcare, and social care organisations in the NHS (Fig. 1), each of whom may unilaterally decide which recommendations they choose to implement or not. Given that the care pathway runs across multiple organisations in one locality and there are multiple independently functioning localities, such an approach inevitably results in more variation in care and inefficient and ineffective care compared with the care outlined in the guideline unless the implementation of NICE clinical guidelines was mandatory. Nationally, there are no clear strategies for their implementation and no organisation is responsible for their implementation, jointly or separately. Policy in mental health is also extremely complex and might contradict itself, e.g. the draft Bipolar Disorder Quality Standards 2015, document refers to 18 other policy documents for the reader to consider at the same time [10]. NICE itself confines its implementation efforts to its own website and a minimal workforce who work regionally on implementation of all their guidelines. NICE has developed many resources that may help the implementation of guidelines, such as guides for commissioners, and have also considerably improved the internal consistency of the guidelines so that they are increasingly compatible with each other and with other NHS policies.

Research on the implementation of any innovation provides few consistencies. A meta-analysis of randomised controlled trials of tailored approaches to the implementation of single interventions taking into consideration the local context of care by involving local practitioners and service users, showed evidence of benefits to patients [60]. A locally-tailored approach systematically identifying and addressing barriers and drivers to the implementation of NICE clinical guidelines has shown promise [61]. However, the evidence on the cost effectiveness of these implementation approaches towards NICE clinical guidelines across a whole clinical pathway is lacking. One approach to achieving such implementation at a local level has been involving networks of professionals and service users working together under umbrella organisations such as the National Institute of Health Research Collaborations for Leadership in Health Research and Care, Academic Health Science Networks, and strategic clinical networks. Implementing evidence-based care in the local context so that it is effective and efficient through these networks can be experienced as an enjoyable challenging and productive experience [62, 63]. Such approaches require training and support, for instance, through knowledge brokers with implementation and quality assurance expertise working alongside teams of clinicians, service users, commissioners, and managers [64].

## Summary

The mandatory implementation of NICE clinical guidelines as the minimum standard of care offers the prospect of delivering high quality care at reduced cost while promoting equity of access and respect for patient and carers’ views and choices, provided the guideline is seen as the starting basis for good clinical care rather than constraining patient-centred care and are kept up to date and trustworthy. Although such an approach is logical based on how the clinical guidelines are derived, there is a limited amount of evidence of economic benefit of the implementation of evidence-based guidelines for bipolar disorder.

## Box 1. Quality Standards for Bipolar Disorder 2015

1. Adults presenting in primary care with depression are offered a referral for a specialist mental health assessment if they have experienced overactivity or disinhibited behaviour lasting 4 days or more.
2. Adults with bipolar disorder have their early warning symptoms and triggers of relapse, preferred response during relapse, and personal recovery goals identified in their care plan.
3. Adults with bipolar disorder are offered psychological interventions specific for their disorder (developmental standard).
4. Adults with bipolar disorder prescribed lithium have their plasma lithium levels maintained at 0.6–0.8 mmol per litre.
5. Women (of childbearing potential) are not offered valproate to treat bipolar disorder unless other treatments are ineffective or not tolerated.
6. Adults with bipolar disorder have a physical health assessment at least annually.
7. Adults with bipolar disorder who currently work, and those who wish to find or return to work, receive supported employment programmes.
8. Carers of adults with bipolar disorder are involved in care planning, decision-making, and information sharing about the person as agreed within the care plan.

## Box 2. Potential benefits of mandatory implementation of NICE Clinical Guidelines in bipolar disorder

1. Establish benchmarks and standards of care for professionals and NHS-funded healthcare.
2. Improve outcomes by promoting interventions of benefit and discourage ineffective interventions.
3. Reduce variation in care and one source of variability in outcome in bipolar disorder which already has a variable course due to its natural history.
4. Encourage collaborative care and continuity of care across NHS organisations through greater clarity about care pathway.
5. Reduced cost of care through greater consistency, increased efficiency, and fewer avoidable adverse incidents.
6. Inform patients, carers, and the public about health conditions and care to inform their decision-making when self-managing, or seeking or discussing care with health professionals.
7. Encourage health professionals to offer personalised care based on recovery principles, i.e. based on what the patient values.
8. Inform public policy.
9. Support quality improvement activities.
10.  Improve quality of training of workforce in both how and what care is delivered.
11.  Focus by NHS and professional organisations on implementing evidence-based care in local and personalised contexts.
12.  Improve quality of NICE clinical guidelines through greater stakeholder engagement in the development of the guideline.
13.  Greater expression of uncertainty in guideline and improved targeting of applied research to address uncertainty.
14.  Greater clarity for industry on how technology may improve clinical care within the NHS by increasing consistency of care, and clarifying areas of uncertainty in delivering care.

## Box 3. Potential harms of mandatory implementation of NICE Clinical Guidelines in bipolar disorder

1. Poor recommendations because scientific evidence is lacking, misleading or misinterpreted [38].
2. Recommendations influenced by personal opinion of key members of clinical guideline, professional interest, financial or professional conflict of interest [12, 57].
3. Lack of consistency in recommendations between national clinical guidelines for the same conditions [39].
4. Institutionalise delivery of ineffective, harmful, or wasteful interventions [38].
5. Professionals and NHS organisations unfairly judged by quality standards or other measures that are not in their control [38].
6. Uncertainty over cost and impact of NICE clinical guidelines if there are unanticipated effects; costs may sometimes increase if, for instance, more patients are referred to secondary care without any improvement in outcome [38].
7. Perceived threat to independence of health professionals and their ability to personalise care of people with atypical presentations [38, 50].
8. Perceived threat to patient-centred care because clinical guidelines are thought to predetermine clinical decision-making without consideration of patient preference [38].
9. Professional disagreement over the nature of evidence underlying clinical decision-making [57, 65].
10. NICE guidelines discount learning from clinical practice and non-random controlled trial evidence on interventions that nevertheless may be informative for practice [66, 67].
11. Increased demand for training, time and other resources among health professionals on how to use and what to deliver in NICE clinical guidelines [68, 69].
12. Lack of consideration of comorbidity, atypical presentation, and clinical uncertainty since clinical guidelines cannot predict every clinical situation [38].
13. Discourage individual innovation that is inconsistent with NICE clinical guideline care except in research [70].
14. Complexity of information in NICE clinical guidelines seen as a potential barrier to obtaining care by some patients who lack confidence or trust in dealing with NHS professionals or organisations [71].
15. Relative absence of research to understand why some clinicians seem to ignore clinical guidelines so a mandatory requirement for implementation may not be effective [50, 72].
16. Mandatory implementation of guidelines may open NHS bodies to additional legal challenges and complaints if NICE clinical guidelines are not followed by them.

Note. Sources for each statement given by reference.

## Abbreviations

NHS: National Health Service
NICE: National Institute of Healthcare and Clinical Excellence
NIHR: National Institute of Health Research
